# Navigating hospitals safely through the COVID-19 epidemic tide: Predicting case load for adjusting bed capacity

**DOI:** 10.1017/ice.2020.464

**Published:** 2020-09-15

**Authors:** Tjibbe Donker, Fabian M. Bürkin, Martin Wolkewitz, Christian Haverkamp, Dominic Christoffel, Oliver Kappert, Thorsten Hammer, Hans-Jörg Busch, Paul Biever, Johannes Kalbhenn, Hartmut Bürkle, Winfried V. Kern, Frederik Wenz, Hajo Grundmann

**Affiliations:** 1 Institute for Infection Prevention and Hospital Epidemiology, University Medical Center Freiburg, Medical Faculty, University of Freiburg, Freiburg, Germany; 2 Institute of Medical Biometry and Statistics, University Medical Center Freiburg, Medical Faculty, University of Freiburg, Freiburg, Germany; 3 Institute of Digitalization in Medicine, University Medical Center Freiburg, Medical Faculty, University of Freiburg, Freiburg, Germany; 4 Public Health Office, Public Health District Freiburg, Breisgau-Hochschwarzwald, Freiburg, Germany; 5 Department of Orthopedics and Trauma Surgery, University Medical Center Freiburg, Medical Faculty, University of Freiburg, Freiburg, Germany; 6 Department of Emergency Medicine, University Medical Center Freiburg, Medical Faculty, University of Freiburg, Freiburg, Germany; 7 Department of Medicine III, Medical Intensive Care, University Medical Center Freiburg, Medical Faculty, University of Freiburg, Freiburg, Germany; 8 Department of Anesthesiology and Critical Care, University Medical Center Freiburg, Medical Faculty, University of Freiburg, Freiburg, Germany; 9 Department of Medicine II, Infectious Diseases, University Medical Center Freiburg, Medical Faculty, University of Freiburg, Freiburg, Germany; 10 Chief Medical Officer, Chairman of the Board of Directors, University Medical Center Freiburg, Medical Faculty, University of Freiburg, Freiburg, Germany

## Abstract

**Background:**

The pressures exerted by the coronavirus disease 2019 (COVID-19) pandemic pose an unprecedented demand on healthcare services. Hospitals become rapidly overwhelmed when patients requiring life-saving support outpace available capacities.

**Objective:**

We describe methods used by a university hospital to forecast case loads and time to peak incidence.

**Methods:**

We developed a set of models to forecast incidence among the hospital catchment population and to describe the COVID-19 patient hospital-care pathway. The first forecast utilized data from antecedent allopatric epidemics and parameterized the care-pathway model according to expert opinion (ie, the static model). Once sufficient local data were available, trends for the time-dependent effective reproduction number were fitted, and the care pathway was reparameterized using hazards for real patient admission, referrals, and discharge (ie, the dynamic model).

**Results:**

The static model, deployed before the epidemic, exaggerated the bed occupancy for general wards (116 forecasted vs 66 observed), ICUs (47 forecasted vs 34 observed), and predicted the peak too late: general ward forecast April 9 and observed April 8 and ICU forecast April 19 and observed April 8. After April 5, the dynamic model could be run daily, and its precision improved with increasing availability of empirical local data.

**Conclusions:**

The models provided data-based guidance for the preparation and allocation of critical resources of a university hospital well in advance of the epidemic surge, despite overestimating the service demand. Overestimates should resolve when the population contact pattern before and during restrictions can be taken into account, but for now they may provide an acceptable safety margin for preparing during times of uncertainty.

The coronavirus disease 2019 (COVID-19) pandemic poses a public health threat, which, if unmitigated, can rapidly overwhelm health care systems.^1–3^ In particular, the demand of patients that require ventilator support becomes critical when available ICU capacities are exceeded^4^. Therefore, nonpharmaceutical interventions have been implemented to ameliorate demand at the peak of the epidemic (the so-called flattening of the curve).^5–7^ National or international measures such as border closures, social distancing, lockdowns, and furloughs have their merit in slowing the epidemic,^8,9^ but they also interrupt global supply chains and may thus prevent healthcare systems from obtaining necessary equipment.^10^

Acute-care hospitals, and especially tertiary-care hospitals, are advised to increase their capacity (ie, beds, personnel, and equipment) well in advance to cope with the expected numbers of COVID-19 patients with severe and critical conditions.^11^ Although some well-known examples show that this can be achieved by creating “new” beds in temporary, purpose-build structures,^12,13^ it most often is accomplished by freeing up existing bed capacity. However, this often occurs at the expense of hospital beds for non–COVID-19 patients,^14^ and it may carry opportunity costs and a protracted burden of disease. Therefore, a timely estimate of the required capacity to treat COVID-19 patients is critical for the planning of sufficient hospital capacity for both COVID-19 and other patients.

The typical course of an epidemic makes early predictions about the volume and timing of peak incidence difficult due to the lack of reliable local data at a time when forecasting and planning becomes crucial. Here, we describe how monitoring of antecedent allopatric epidemic waves, combined with timely local estimates, and continuous monitoring between March 15 and April 28, 2020, helped a university tertiary-care center in southwestern Germany prepare for the pandemic as well as scale up its bed capacity. For hospital management, we offer a forecast strategy within defined credibility boundaries, allowing for better bed planning, allocation, and procurement of essential resources.

## Methods

The University Medical Center Freiburg (Universitätsklinikum Freiburg, UKF) is a 1,600-bed tertiary-care center; it is the largest regional hospital in southwestern Germany. As an acute-care hospital, it draws patients from ~60% of the Freiburg, Breisgau, and Hochschwarzwald health districts. As a tertiary-care referral and trauma center, UKF serves other district hospitals in the Upper Rhine region that borders Switzerland to the south and the French Alsace, Departments Haut-Rhin and Bas-Rhin, to the west.

COVID-19 cases were defined as symptomatic individuals with RT-PCR positivity for severe acute respiratory coronavirus virus 2 (SARS-CoV-2) ascertained at 1 of 3 accredited diagnostic laboratories. Tests were carried out at community diagnostic centers, by general practitioners, or on admission to the UKF. Positive results were reported to the district health authorities according to German notifiable disease law and were recorded as COVID-19 on hospital admission in electronic patient records. For COVID-19 patients, dates of admission, between-ward referrals, and discharge were kept in electronic patient records available through the hospital’s patient administration system.

Prediction of the expected bed demand at the UKF was initially constrained by the availability of valid and representative data. Therefore, we used a 2-stage approach to model the expected incidence of COVID-19 patients among the UKF catchment population. In the first stage, we used a static incidence model based on extrapolations of antecedent allopatric epidemic waves, and we parameterized a hospital care-pathway model using a panel of experts. In the second stage, we used a dynamic incidence model, informed solely by the number of confirmed cases for the Freiburg, Breisgau, and Hochschwarzwald health districts, and we parameterized the care-pathway model using the individual electronic patient records as documented by the UKF hospital patient administration system.

### Static incidence model

To provide a forecast prior to the local epidemic surge, we analyzed aggregated data from Italy and Germany as reported by John Hopkins University,^15^ as well as subnational data for the Lombardy region and the Lodi province, available through the website of the Italian Dipartimento della Protezione Civile.^16^ We calculated the delay between the cumulative per capita incidence in each region relative to the Italian epidemic, and we normalized the epidemic curves by correcting for this delay, thus overlaying and combining all curves into a single epidemic trajectory. We tested the trajectory for saturation properties and decided on a tentative epidemic peak on which we mirrored the epidemic curve, following a symmetry conjecture.

To predict the Freiburg regional epidemic, we calibrated the epidemic curve to the UKF catchment population (taking into account observed delays) by multiplying the per-capita values with the catchment population size of the UKF. Calculation of catchment size was based on the relative number of admissions to the UKF compared to all other hospitals in the state of Baden-Württemberg (11 million inhabitants) taken from a comprehensive data set recording all patient admissions on an annual basis. The database was made available by the largest health insurer (Allgemeine Ortskrankenkasse Baden-Württemberg).

### Dynamic incidence model

We produced local data–informed estimates using the dynamic incidence model after cumulative case counts reached 1 per 1,000 population in the Freiburg, Breisgau, and Hochschwarzwald health districts (492,000 total inhabitants). We imputed the likely date of infection for each COVID-19 case in the health district and estimated the time-varying effective reproduction number (R_T_)^17^ based on the probability distribution of the serial intervals between consecutive case generations.^18^ We used an individual-based stochastic simulation to predict the local future incidence, assuming that R_T_ declined exponentially over time, fitting R_f_(t) = ae^bt^ to the estimated R_T_. This declining function serves as a phenomenological approximation to the observed changes in R_T_ and is used to calculate the transmission parameter of the SIR model (see Supplementary Text S1 online).

### Care pathway model

**Fig. 1.**
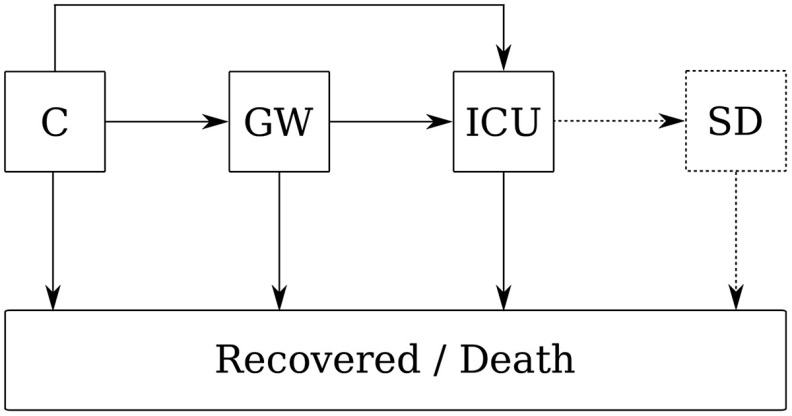
Model structure. The COVID-19 care pathway describes how patients progress from confirmed cases in the community (C), to be admitted on general wards (GW), to intensive care units (ICU), and to step-down units. Some COVID-19 patients are admitted directly to the ICU from the community. The step-down unit was only included in the agent-based model.

To convert the forecasted regional incidence to bed demand, we created an agent-based model for the in-hospital care pathway (Fig. 1) consisting of confirmed cases, equal to the results of the above incidence models, patients admitted to general wards, and patients admitted to intensive care units (ICUs). Patients are assumed to follow 1 of 3 possible tracks through the hospital: (1) admitted to and discharged from general wards; (2) admitted to general wards, moved to ICUs, and then discharged from an ICU; or (3) directly admitted to an ICU and discharged from there. Within the model, we make no distinction between discharge, death, and removed patients as end of stay. The model thus contains 5 parameters: the distributions of the length of stay in general wards and ICUs, the distribution of time from infection to hospital admission, the proportion of patients admitted to hospital, and the proportion of patients directly admitted to an ICU.

In the first stage, we used a consensus care pathway parameterized by judgment of a panel of experts. In the absence sufficient local data, we asked 4 consultant experts (3 intensivists and 1 infectious diseases specialist) to make estimates about the expected care pathway of COVID-19 patients during their treatment in the UKF.

Once the number of admitted COVID-19 patients had surpassed 150, we parameterized the empirical care pathway using the individual electronic patient records as documented by the UKF hospital patient administration system. Based on these observations, we added a fourth compartment for those patients who left the ICU and returned to a general ward and named this a step-down unit.

We analyzed the time until the end of stay in each compartment split between those patients being transferred to another compartment and those ending their stay. We fit both exponential and Weibull distributions to the hazard functions of leaving each compartment. These served as the main parameters in the empirically informed care pathway. Furthermore, we calculated the admission rate as the cumulative number of admissions divided by the cumulative number of confirmed cases, and similarly, we determined the proportion of patients being directly admitted to the ICU.

## Results

**Fig. 2.**
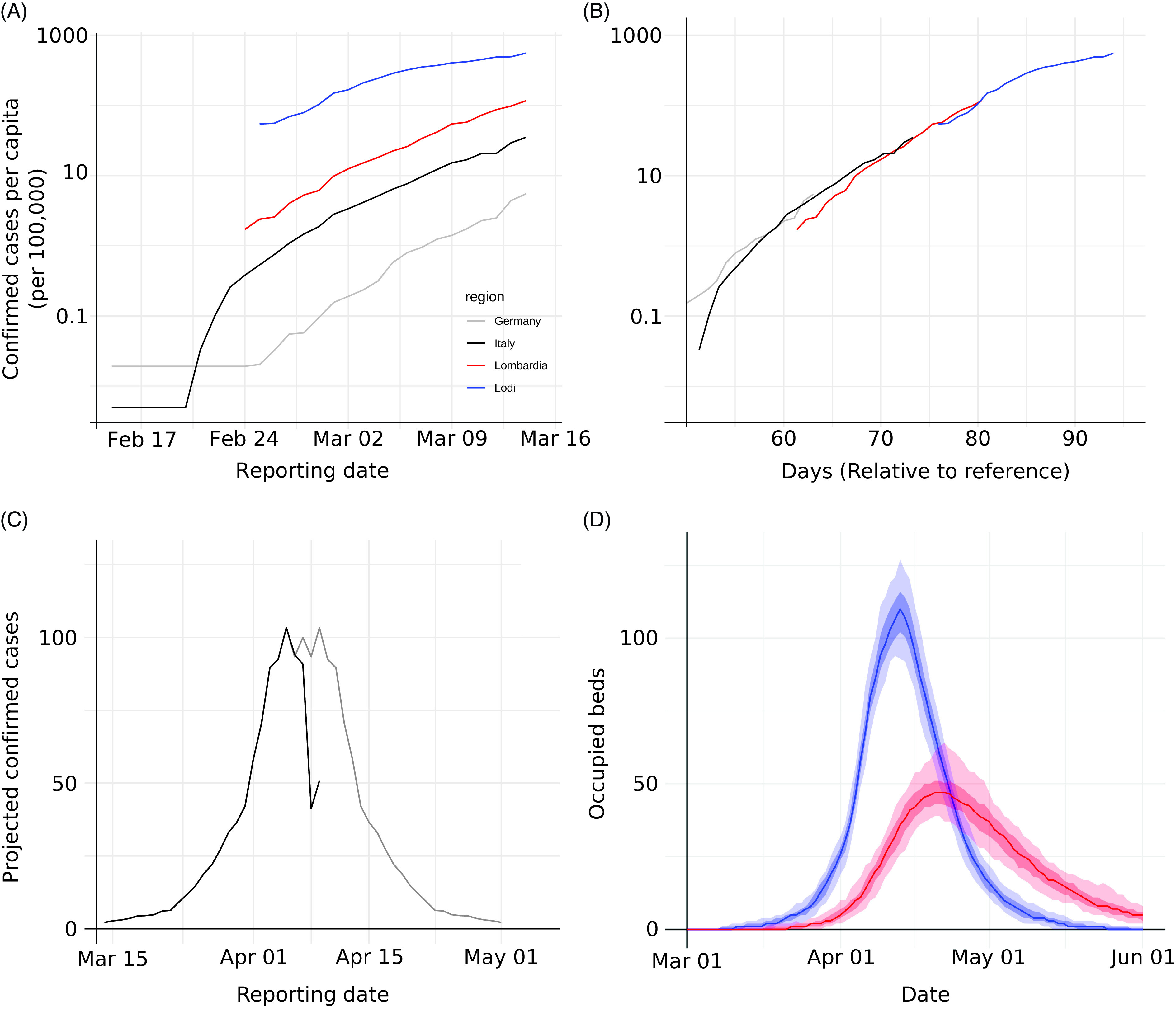
Early forecast using the static model. (A) The trajectory of the number of confirmed cases in Germany, Italy, Region of Lombardy, and Province of Lodi were (B) normalized and projected as a single curve by compensating for the apparent delay between locations, and (C) the downward slope (grey) was predicted assuming a symmetry conjecture of the observed upward slope (black). (D) Expected bed occupancy for the UKF (catchment size 290,000 people; general wards in blue and ICU in red). Light shades indicate 95% CI and dark shades indicate interquartile ranges. Predictions are based on the COVID-19 care pathway using expert consensus. Bed demand peaked on the general wards at 116 beds on April 9 and in ICUs at 47 beds on April 19.

During the first 2 weeks of March 2020, the Lombardy region of Italy and the Departments Haut-Rhin and Bas-Rhin in France saw a rapid expansion of the COVID-19 epidemic.^1,19,20^ The civil protection authorities in Italy reported confirmed cases on a daily basis. We observed that cumulative case counts for Germany followed the same exponential trajectory as the Lodi province, the Lombardy region, and Italy as a whole, albeit with some delay but similar growth rates (Fig. 2A and B). The delay between Germany and Italy was estimated to be 10 days, while the Lodi province was 21 days ahead of Italy. Only the trajectory for Lodi showed an obvious and sustained slowing of the growth rate prior to March 15 (Fig. 2A and B). We chose March 7 as the saturation point for the epidemic in Lodi province, and assuming an equivalent dynamic, the peak for Germany could be projected to occur on April 7. Applying the combined trajectory (Fig. 2C) to the catchment population of the UKF (290,000 people), we expected 103 incident cases on the day of the epidemic peak.

To predict the expected bed and ventilator demand, we chose to describe the expected COVID-19 patient care pathway on the basis of expert knowledge and opinion. The expert estimates were in general agreement on most of the care-pathway parameters (Supplementary Table S2 online). We combined the assessments by averaging the individual parameter estimates of all 4 experts into a single consensus care pathway. Combining the care pathway with the results of the static incidence model, we predicted the demands for general ward and ICU beds to peak on April 9 (116 beds) and April 19 (47 beds), respectively (Fig. 2D).

**Fig. 3.**
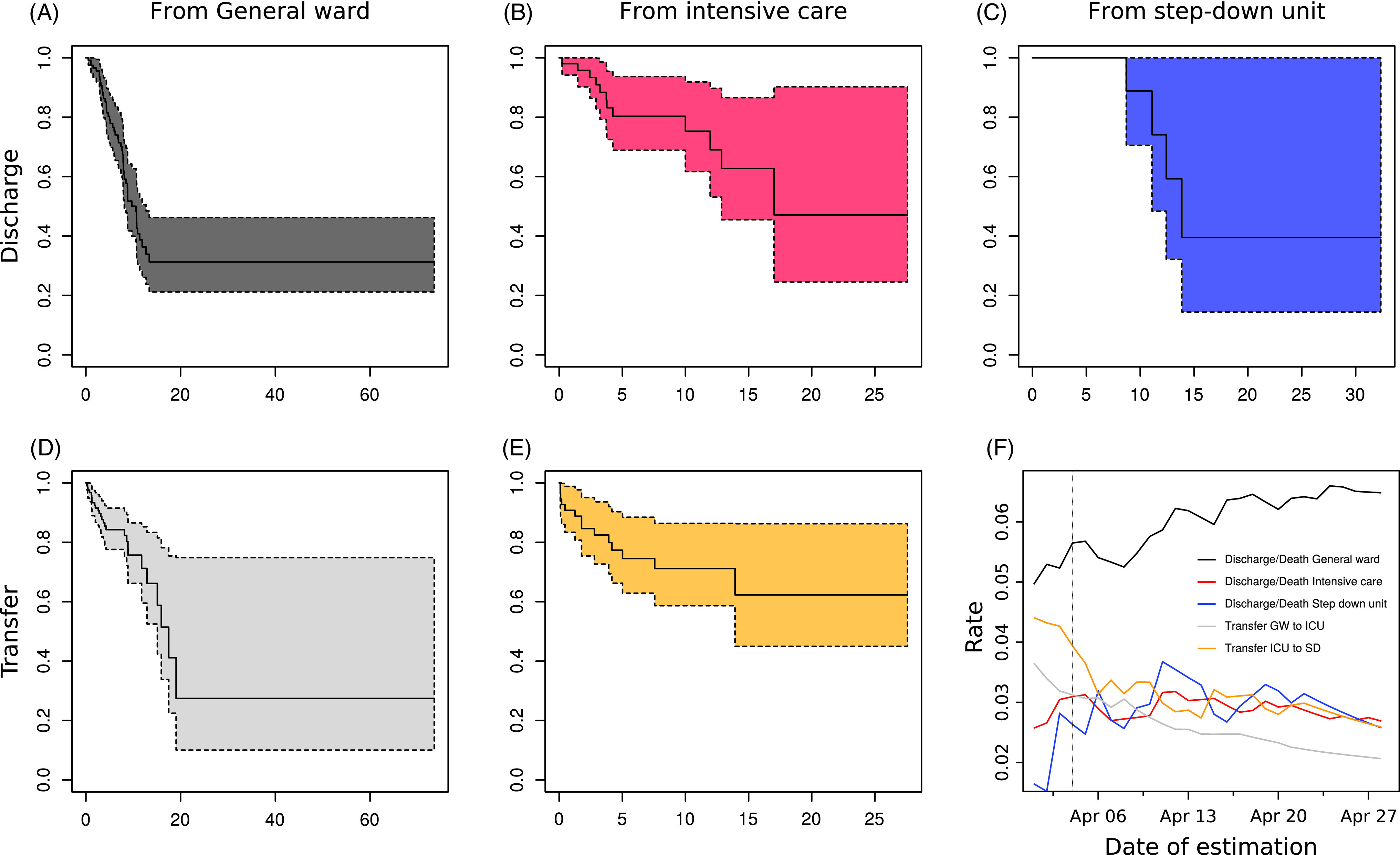
Survival analysis (A–E) Kaplan-Meier estimators for the stay on the general ward (A and D), ICU (B and C), and step-down unit (C), for patients that are discharged (A, B, and C) and transferred to the following ward (D and E), based on the data observed on April 5. (F) The estimated rates of discharge, death, and transfers over time, based on continuously accumulating data.

By April 5, the availability of locally generated data provided the opportunity to populate the care-pathway model with empirical local data and to offer the first predictions using the dynamic incidence model. At the time, 153 patients had been admitted to the UKF, of whom 55 required ventilator support on ICUs. Also, 28 were admitted directly to an ICU, whereas 27 had a prior stay on a general ward. Furthermore, 14 had already been transferred to the step-down unit. Of all admitted patients, 87 were still hospitalized (general ward, n = 48; ICU, n = 29; and step-down unit, n = 10). Based on the estimated hazard of end of stay on a general ward (0.05678 d^-1^) and the hazard of transfer to an ICU (0.0307 d^-1^), we estimated that patients spent a mean of 11.4 days on a general ward (Fig. 3A and D). Similarly, we estimated patients stayed on average 14.7 days on an ICU (end of stay hazard, 0.03127; transfer hazard, 0.03648) (Fig. 3B and E), and 40.5 days on a step-down unit (Fig. 3C, based on 4 discharges). The hazard estimates stabilized over time as more data were added (Fig. 3F and Table S3 online).

**Fig. 4.**
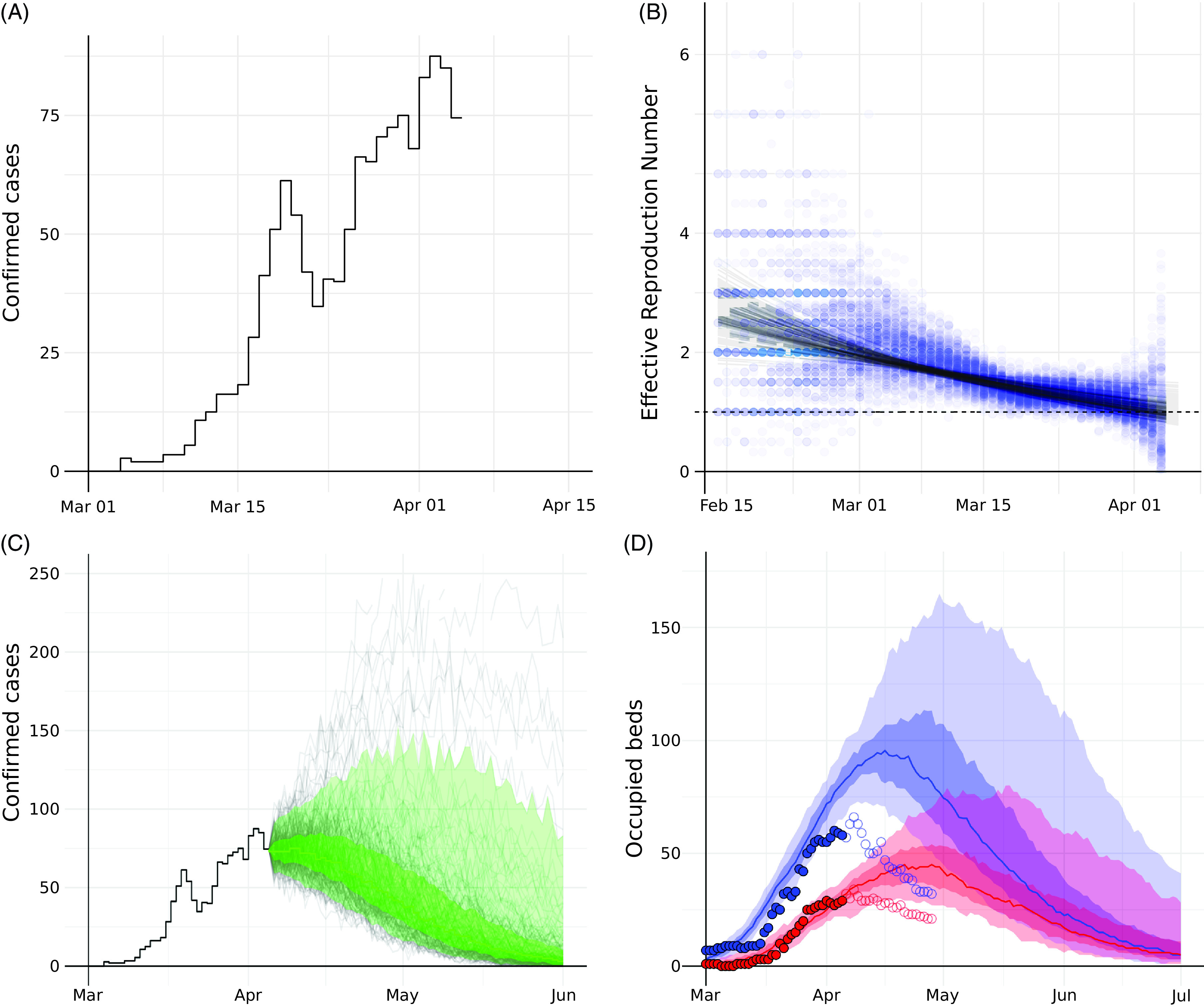
Late forecast using the dynamic model based on locally available data on April 5. (A) The observed incidence of confirmed cases in the Freiburg, Breisgau, and Hochschwarzwald health districts combined. (B) Backward model: Estimates of the time-varying Reproduction number (blue dots) over 100 stochastic simulations, with fitted R_f_(t) trajectories (black lines). (C) Forward model: Forecasted incidence of confirmed cases, grey lines show single simulation results, green line show the median, with green shade showing the interquartile range and green light shade 5%–95% of the simulation results. (D) Estimated bed demand (median, IQR, 5%–95% range) for the general wards (blue) and ICU (red). Circles denote actual observed number of beds occupied (closed: past days; open: future days not known at the time of the analysis on April 5).

On the same day, 1,372 cumulative cases (Fig. 4A) had been reported in the health district (2.8 per 1,000 inhabitants). Since the onset of the epidemic, the time-varying reproduction number (R_T_) showed a clear decline, starting at a median of 3.5 and decreasing to 1.1 (Fig. 4B). The dynamic incidence model projected a peak incidence of a median number of 90 cases for April 8 (Fig. 4C). Although there was considerable variation between model simulations (the lowest peak was projected at 74 cases, the highest at 1,186), 75% of the realizations suggested little or no further increase with an imminent saturation of the epidemic in the near future. Dynamic incidence forecasts could be produced from March 24, albeit without empirical local care-pathway data until April 5. While adding daily reported cases to the dynamic incidence model, iterations generated fluctuations and occasional uncertainty (see Supplementary Fig. S4 online and Supplementary Movie S5 online). The overall trajectory stabilized after April 6. Combining these projections with the empirically informed care pathway, we estimated a peak demand of 102 general ward beds (IQR, 92–121) on April 17 and 49 ICU beds (IQR, 42–58) on April 25 (Fig. 4D). Observed bed occupancy peaked on April 8 for both the general wards (66 beds occupied) and the ICUs (34 beds occupied). By April 14, the forecasted bed demand aligned with the observed occupancy (Supplementary Fig. S4, column 5, online), likely because the incidence model predicted the declining phase of the epidemic curve more precisely.

## Discussion

During the current COVID-19 pandemic, hospitals have reported a breakdown of services when the surge of patients in need of treatment and ventilator support outpaced available capacities.^21^ Early predictions about the timing and volume of maximum service demand, (ie, expected general ward and ICU bed occupancy) are therefore critical in the early stages of an epidemic. These predictions may help guide the upscaling of a hospital’s bed capacity, redistributing personnel, and/or storage of crucial equipment. However, data that could provide a basis for predictions are often equivocal or insufficient in the incipient stages of an epidemic. In an attempt to decrease these uncertainties, we have utilized allopatric and locally available data for contingency planning for healthcare authorities and hospital management.

We took advantage of antecedent epidemic waves in neighboring countries, especially in Northern Italy, where daily case counts were available at the level or provinces with population sizes comparable to our own health district. When we normalized the epidemic trajectories between nations, regions, and provinces, we found that uncontrolled transmission in populations with similar contact patterns^22^ was comparable during early stages of epidemics. Therefore, we informed our local predictions by a static model calibrated by the per capita case counts and delay of onset in Lodi, Lombardy, and Italy, assuming that local epidemiology would be similar to that in Northern Italy.

This static forecast gave local healthcare authorities and hospital management of the UKF 3 weeks to prepare for the expected number of COVID-19 patients at the epidemic peak, ample time to call off elective interventions, upscale ICU capacity, reallocate staff, take stock, and strengthen efforts to procure essential equipment such as disposables, personnel protective equipment (PPE), oxygen, etc. Given the size of the epidemics in Lombardy and the neighboring department in France, the results of the static model were used as the lower bound of the required capacity. Whether regional district hospitals would be able to cope with the likely caseloads in the larger region was uncertain. The entire region consists of a catchment population 3 times the size of the UKF’s catchment (1 million vs 290,000), which was forecasted to result in an additional demand for 284 general ward beds and 121 ICU beds in the surrounding hospitals (Supplementary Fig. S6 online).

The dynamic model required locally available data. After March 24, sufficient numbers of incident cases had been ascertained and moved through the UKF, allowing forecasts to be updated on a daily basis. This real-time tracking provided us with the potential to adjust hospital planning. In particular, it provided the means to fine-tune ICU and general ward capacities and to reconcile foreseeable equipment demands with available stock and expected deliveries. In hindsight, our model predicted the peak to be higher and later than the peak observed, but no further upscaling of capacity was required.

The implicit continuity assumption of the parameters made our dynamic prediction models vulnerable to unforeseen changes in transmission dynamics, such as the introduction of nonpharmaceutical interventions, or changes in the ascertainment of confirmed cases. For Freiburg and surroundings, restrictions took effect on March 20 and included closures of schools, shops, and restaurants; prohibition of large gatherings; and an obligation for social distancing. Therefore, our fitted trajectory to the estimated time-varying reproduction number (R_T_) may initially have been too high because preintervention R_T_ estimates still carried too much weight. Additionally, random mixing assumed in the standard SIR model overestimates epidemic growth because extant contact patterns are generally assortative. Furthermore, the incidence model assumes homogeneity in the patient population, that is, each infected individual having the same probability of being admitted to hospital. In reality, risks are strongly associated with age,^23-25^ which skews hospitalization rates depending on demographic composition. We therefore suggest that improved prediction models should account for age-stratified contact patterns, age structure of hospital catchment populations, and the effect of nonpharmaceutical interventions.

Local data–based, short-term forecasts of hospital admissions are vital to epidemic planning by hospitals because the onset of epidemics may vary greatly between different geographical regions. Also, local bed demand may peak at different times. The lack of local data in the early phase of an epidemic is challenging in this respect. We solved this issue by forecasting in 2 stages, with an early “crude” estimate based on observed COVID-19 outbreaks abroad and a later, continuously adjusted, nowcasting and forecasting using locally ascertained data. Thus, we were able to navigate hospital capacities by setting weekly targets while adjusting the elective admission and discharge policies according to the most current epidemic situation.

## Supplementary material

For supplementary material accompanying this paper visit http://dx.doi.org/10.1017/ice.2020.464. click here to view supplementary material
