# Supplementary material for: Navigating hospitals safely through the COVID-19 epidemic tide: Predicting case load for adjusting bed capacity
Source: Infect Control Hosp Epidemiol. 2020 Sep 15:1–6. doi: 10.1017/ice.2020.464 (PMC8160497; doi:10.1017/ice.2020.464)
Supplement: Supplementary file 1 [file S0899823X2000464Xsup001.docx]

**Navigating hospitals safely through the COVID-19 epidemic tide: predicting case load for adjusting bed capacity.**

*Tjibbe Donker, Fabian Bürkin, Martin Wolkewitz, Christian Haverkamp, Dominic Christoffel, Oliver Kappert, Thorsten Hammer, Hans-Jörg Busch, Paul Biever, Johannes Kalbhenn, Hartmut Bürkle, Winfried Kern, Frederik Wenz, Hajo Grundmann*

**Supplementary information**

***Text S1:*** Supplementary methods

***Table S2:*** expert panel estimates of COVID-19 patient care paths

***Table S3:*** Fitted hazard functions, for each day between day April 5 and April 28.

***Figure S4:*** Estimates produced each day between March 24 and Apri 28, showing incidence (1^st^ column), Rt estimate (2^nd^ column), incidence forecast (3^rd^ column), bed demand forecast based on expert estimates (4^th^ column), and bed demand forecast based on observed COVID-19 patient care-paths (5^th^ column). Full color illustrations in change on April 5 from column 4 to 5 indicating the switch from expert consensus care-path to observed care-path.

***Movie S5:*** Estimates produced each day between March 24 and Apri 28, similar as Figure S4, in movie format.

***Figure S6:*** *Early forecast using the static model for the extended region of Freiburg, including the catchment population of hospitals expected to refer patients to the UKF when their capacity is reached (total size: 1 million inhabitants). Expected bed occupancy, (general wards in blue, ICU in red). Light shades 95% CI, dark shades interquartile ranges. Predictions are based on The COVID-19 care path using expert consensus. Bed demand peaked on the general wards at 116 beds on April 9 and in ICUs at 47 beds on April 19.*

**Supplementary text S1.**

**Stochastic simulation**

The model has two major components: the incidence model and the care-path model of patients moving through the hospital. The incidence model is used to forecast the epidemic curve in the immediate catchment population of the UKF (290.000 people in the health districts of Freiburg / Breisgau-Hochschwarzwald), while the care-path model converts the incidence into the expected bed demand in the hospital over time. We used stochastic, agent-based approaches for both the incidence model and the within-hospital model. We resorted to this class of computational models because the number of expected cases was expected to be relatively low (in comparison to those in an entire country). Stochastic fluctuations may therefore cause potentially large effects.

***Backward imputation: time of infection***

We assumed that the delay between the time of infection and reporting is exponentially distributed with a mean length of 7 days. For each case *I_i,t_* infected on day *t*, we we assigned a reporting delay *t_d_* from the delay distribution, thus assigning it a day of infection *d:*

*I_i,d_=I_i,t_ – t_d_*

The number of cases infected on day *t* is thus:

$I_{y,t}=\sum_{i}^{d=t} I_{i,d}$

We then determined the number of non-reported cases (cases that have already been infected but have not been reported yet) *I_N,t_* for each day *t* using a negative binomial distribution with *N=I_y,t_ and p=p_R_(z),* where p_R_(z) is the probability of having been reported z days after infection, taken from the above exponential reporting delay distribution.

*I_A,t_ =I_y,t_+ I_N,t_*

***R_t_ Estimation***

To calculate the time-varying reproduction number, we adopted an individual-based version of the Wallinga&Teunis methods^17^. For each case we assigned a day of infection based on the serial interval and the day of infection of past cases. The likelihood of a case *i* having been infected by case *j* a given a time *(t_i –_ t_j_)* between them, is based on the serial interval distribution and normalised for the chance of case *i* having been infected by any other case. (Following naming convention by Wallinga&Teunis). We used a gamma distributed with parameters α=1.87 and β=0.28 as serial interval, as estimated from observed infection pairs in Italy^18^.

$P_{i,j}=\frac{w\left( t_{i}-t_{j} \right)}{\sum_{i}^{i\neq k} w\left( t_{i}-t_{k} \right)}$

We selected for each case a random infector based on this likelihood, tracked the number of cases infected by each possible infector (i.e. the infector’s reproduction numbers) and averaged the reproduction numbers per day. We fitted a declining exponential function to the estimated *R_t_*:

$R_{f}\left( t \right)=ae^{-bt}$

This function was used as input for the transmission process in the forward SIR model.

**Forward model: incidence forecast**

The forward incidence model was based on a standard SIR compartment model, with individuals being either susceptible (*S*), infectious (*I*), or removed (*R*). However, instead of modelling the compartments directly, we track the infection events by day and calculate the size of the compartments based on the observed events.

The total population size is set at *N_p_*, and the number of incident infectious individuals at day *t* is *I_t_*, and under the assumption that infection causes immunity, the number of individuals still susceptible to infection is

$S_{t}=N_{p}-\sum_{i=0}^{t} l_{i}$.

By definition, the time between new cases arise from previous cases is distributed according to the serial interval distribution (Here taken as a gamma distribution with parameters α=1.87 and β=0.28^18^). For each day, we therefore calculate the number of source infections underlying potential new infections:

$M_{t}=\sum_{i=1}^{t-1} I_{\left( t-i \right)}w\left( i \right)$

which is the total number of previous incident cases times the probability that the serial interval between them and a secondary case equals the number of days ago they were infected. The transmission potential from one individual to any other in the population is given as

$\beta_{t}=\frac{R_{f}\left( t \right)}{N_{p}}$ ,

which means that the probability for a single individual to become infected is

$p_{I,t}=1-\left( 1-\beta\right)^{M_{t}}$

The number of expected new cases on day t is then given by a binomial distribution with parameters *n=S_t_* and *p=p_I_* determines the probable number of cases being newly infected on day *t*.

Note that the mean expected value of the number of new cases is *n p*, which can be rewritten as *S_t_ p_I_ for this model*. If the epidemic is in its early stages, with a single infected case, $M_{t}=\sum I_{\left( t-i \right)}w\left( i \right)=1$, and $p_{I,t}\approx\beta_{t}$and *S_t_=N_p_-1*. Rewriting *p_I,t_* to$p_{I,t}\approx R_{f}\left( t \right)/{N_{p}}$, we see that the expected number of new cases arising from a single first case $N_{p}R_{f}\left( t \right)/{N_{p}}=R_{f}\left( t \right)$.

**Within-hospital**

Per day, we determine the hospitalization status for each individual incident case in the community. When hospitalized, each of the cases may have moved 1) to a general ward, 3) to an ICU, or 4) removed from hospital. We picked each track according to the input probabilities (*a_H_, a_I_, a_N_*, respectively).

For the cases in the hospitalized tracks, we picked the event times using the fitted hazard functions to the length of stays in the respective wards. For patients in general wards and in ICUs, we picked both a time of discharge (or death) and a time of transfer to the next compartment (ICU and step down ward, respectively). Whichever event happened first determined the next step in the care path of the patient.

Per day, we reported the number of patients in each compartment at virtual midnight, after all admissions and discharges for that day had occurred. We added the number of beds occupied on the general ward and step-down unit, because were considered to be the same type (i.e. non-ventilator) beds.

**Survival analysis**

From April 5 onwards, as the number of admitted COVID-19 patients surpassed 150, we estimated the model parameters based on the observed COVID-19 patient care path through our hospital. Based on the observed care pathways at this point, we included a post-ICU stay on the general ward (i.e. the step-down unit: D) into the within-hospital model. Patient admission data were linked with their COVID-19 status in the centralised patient administration system. The COVID-19 status of the patients was defined as a confirmed SARS-CoV-2 infection by RT-PCR, either determined by the hospital laboratory after admission of the patient, or before admission by one of the external laboratories. We assumed that the most recent hospital stay of each COVID-19 positive patient could entirely be attributed to the viral infection, with the admission day being the start of COVID-19 stay.

**Supplementary Table S2:** expert panel estimates of COVID-19 patient care paths

|  | **1 (PB)** | **2 (HB)** | **3 (WK)** | **4 (JK)** | **Expert panel** |
| --- | --- | --- | --- | --- | --- |
| **% admitted to GW first** | 25% | 20% | 20% | 20% | 21.25% |
| **% admitted to ICU directly** | 2% | 3% | 1% | 4% | 2.5% |
| **Length of Stay GW** | 11 days | 5 days | 5 days | 5 days | 6.5 days |
| **% discharged from GW without ICU stay** | 75% | 78% | 75% | 80% | 77% |
| **% moved from GW to ICU** | 25% | 22% | 25% | 20% | 23% |
| **% ICU survivors** | 60% | 80% | 80% | 80% | 75% |
| **LOS ICU survivors** | 14 days | 11 days | 14 days | 14 days | 13.25 days |
| **% Rapid fatal ICU** | 30% | 5% | 5%* | 5%* | 11.25% |
| **LOS Rapid fatal ICU** | 5 days | 5 days | 5 days | 3 days | 4.5 days |
| **% ultimately fatal ICU** | 10% | 15% | 15%* | 15%* | 13.75% |
| **LOS ultimately fatal ICU** | 14 days | 21 days | 21 days | 21 days | 19.25 days |
| **Mean LOS ICU** | 11.3 days | 12.2 days | 14.6 days | 14.5 days | 13.15 days |

***
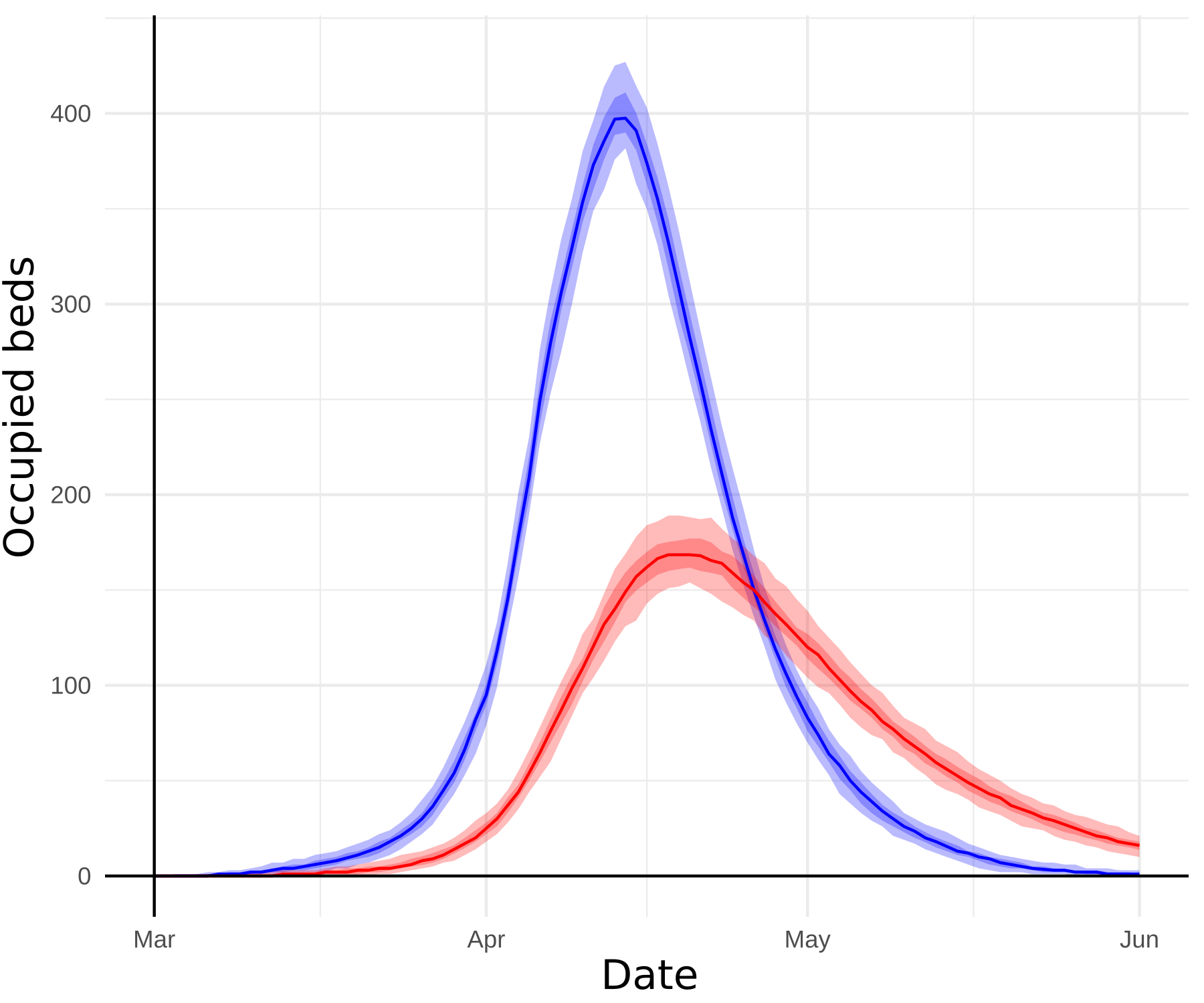
***

***Supplementary figure S6:*** *Early forecast using the static model for the extended region of Freiburg, including the catchment populations of hospitals expected to refer patients to the UKF when their capacity is reached (total size: 1 million inhabitants). Expected bed occupancy, (general wards in blue, ICU in red). Light shades 95% CI, dark shades interquartile ranges. Predictions are based on The COVID-19 care path using expert consensus. Bed demand peaked on the general wards at 400 beds on April 9 and in ICUs at 168 beds on April 19.*
